# Medical Journals

**Published:** 1860-09

**Authors:** 


MEDICAL JOURNALS.—In bringing the third volume of the *New York Medical Press* to a close, the editors announce that its publication will be discontinued. They have been compelled to take this step on account of important business and professional engagements.

Professor Weber has become associated with Drs. E. B. Stevens and J. A. Murphy, in the editorial management of the *Cincinnati Lancet and Observer;* while Drs. Stevens and Murphy hold the same relation to the *Cleveland Medical Gazette.* Notwithstanding this new arrangement, equivalent to a consolidation, each journal will retain its own name, and appear simultaneously at Cleveland and Cincinnati.

